# Patterns of systemic problems in Ghana's poultry value chain: A group model building approach

**DOI:** 10.1016/j.jafr.2025.101738

**Published:** 2025-04

**Authors:** Joshua Aboah, Dolapo Enahoro, Charles Mensah, Nana Adwoa Agyemang, Ebenezer Kondo, Desmond Ayertey

**Affiliations:** a WorldFish, Inclusive Aquatic Food Market Systems Team, Nairobi, Kenya; b International Livestock Research Institute, People, Policies and Institutions Program, C/o IWMI-Ghana, Accra, Ghana; c University of Ghana, Department of Agricultural Economics and Agribusiness, Accra, Ghana; d Ghana Atomic Energy Commission- Biotechnology and Nuclear Agriculture Research Institute, Kwabenya, Accra, Ghana; e Commonwealth Scientific and Industrial Research Organisation, Agriculture and Food Research Unit, Food System Dynamics Team, St Lucia, Brisbane, Australia

**Keywords:** Systems thinking, Chicken, Causal loop diagram, Agricultural policy

## Abstract

Ghana's poultry sector faces different interrelated systemic challenges, often diagnosed in isolation, leading to interventions that neglect unintended consequences across the value chain. Consequently, a holistic prognosis of the impact of these systemic problems that considers the different facets of the poultry industry is required.

This paper aims to examine the system archetypes emerging from the inherent industry-level and farm-level problems in Ghana's poultry sector. Adopting a participatory group model building process, causal loop diagrams and feedback loop analyses were applied to understand the interacting factors in four systemic problems prioritised by stakeholders in Ghana's poultry value chain. Four causal loop diagrams were mapped for these systemic problems; *(i)* inadequate research funding *(ii)* low adherence to biosecurity measures at the farm level; *(iii)* lack of access to credit; and (*iv*) the competition from cheap imports of poultry meat products.

The findings highlight three emerging problem archetypes. First, the underachievement archetype, specifically the limit to growth, emerges when technology adoption due to increased research funding interacts with the non-adherence to biosecurity measures as a cost-cutting strategy at the farm level. Second, the out-of-control archetype emerges when the misuse of antimicrobials due to the non-adherence of biosecurity measures interacts with the industry's collapse and the consequential surge in chicken imports into the country. Third, the relative achievement archetype emerges from the reinforcing feedback loop which centres around the surge in imported chicken as a response to looming food insecurity concerns arising from insufficient domestic supply. The “*success to the successful*” archetype is thus created, where importers in the poultry value chain become more prosperous at the expense of the entire industry.

The paper presents solutions to the emerging problem archetypes, providing stakeholders with a chance to evaluate the unintended consequences of proposed government policies aimed at rejuvenating local poultry production.

## Highlights

• Ghana's poultry sector faces systemic challenges often addressed without considering unintended impacts.
• A holistic approach is needed to understand and address interconnected problems across the poultry value chain.
• Participatory modelling identified four key issues: research funding, biosecurity, credit access, and import competition.
• Three archetypes—underachievement, out-of-control, and relative achievement—emerge from the industry's systemic problems.

## Graphical abstract

**Figure:**
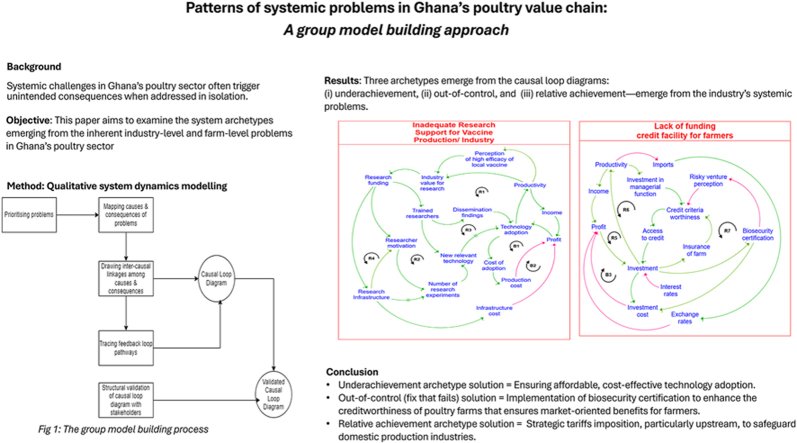

## 1 Introduction

The poultry industry in Ghana has enormous potential to improve the food and nutrition security of households in the country [1]. Chicken meat and eggs consumption in the country has increased in the past two decades [2]. Data from FAOSTAT [3] show a consistent increase in the number of chickens produced and slaughtered for meat in Ghana from 2010 to 2023. Over the 13-year period, the average annual production was 72,892,500 chickens, with a peak of 93,666,000 chickens in 2023. During the same period, the average number of chickens slaughtered was 74,775,786 reaching a maximum of 96,223,000 in 2023. Details of the chicken meat and egg production in Ghana from 2010 to 2023 is presented in Table 1.

**Table 1:** Production of chicken meat and egg in Ghana.

*Year*	*Chicken meat produced*	*Eggs production*	*% change in egg production*	*Slaughtered chicken*	*% change in slaughtered chicken*
2010	47,752,000	873,800,000		49,000,000	
2011	52,575,000	946,400,000	8.31	54,000,000	10.20
2012	57,885,000	950,000,000	0.38	59,200,000	9.63
2013	63,732,000	1,000,000,000	5.26	65,200,000	10.14
2014	68,511,000	1,056,000,000	5.60	70,500,000	8.13
2015	71,594,000	1,136,626,000	7.64	73,200,000	3.83
2016	73,885,000	1,156,353,000	1.74	76,000,000	3.83
2017	75,363,000	1,140,000,000	−1.41	77,000,000	1.32
2018	81,955,000	1,254,041,000	10.00	84,000,000	9.09
2019	89,210,000	1,296,689,000	3.40	91,629,000	9.08
2020	81,769,000	1,231,786,000	−5.01	83,971,000	−8.36
2021	81,487,000	1,231,567,000	−0.02	83,646,000	−0.39
2022	81,111,000	1,230,465,000	−0.09	83,292,000	−0.42
2023	93,666,000	1,231,272,000	0.07	96,223,000	15.52

Despite the growing importance of poultry in Ghana's protein-economy [4], the local poultry industry is bedevilled with systemic challenges intertwined across various facets of the industry including production, epidemiology, and marketing; each exerting its own unique influence and contributing to the complexity of the overarching problems [5,6]. For instance, Enyetornye et al. [7] identified disease outbreaks as a significant challenge in the poultry sector. Agyeman et al. [8] further emphasised the limited access to credit facilities, which hinders poultry farmers' ability to acquire essential inputs and technologies that could enhance productivity. Additionally, Sumberg et al. [6] and Anang et al. [9] highlighted high feed costs as another critical issue facing Ghana's poultry industry. Feed costs constitute a large portion of total production expenses and are vulnerable to fluctuations in global and regional grain prices, which drive up production costs and make locally produced chicken more expensive than imported alternatives. Furthermore, the disconnect between consumers' stated preference for domestic chicken and their actual purchasing behaviour, which tends to favour the more affordable imported chicken is an important marketing challenge [10].

Due to these challenges, domestic production is unable to match the growing consumer demand for chicken meat [2]. While extant studies have examined different aspects of the systemic challenges in isolated cases, drawing insights from the perspectives of individual actors within the value chain [[8], [9], [10], [11]], their efforts often fall short of capturing the holistic scope of systemic problems.

There have been calls for a ban on the importation of chicken into Ghana due to concerns that overreliance on import predisposes the sector to global supply disruptions leading to food insecurity [8,12]. Compared to Senegal, where a ban on poultry imports is in effect, the per capita consumption of chicken in Ghana is 57 % higher [2]. However, the annual chicken production in Senegal is 400 % higher than Ghana's production levels, even though Senegal’s human population is roughly half of Ghana’s. Ghana relies heavily on lower priced imported chicken to meet its growing demand. Also, the cost of feed and day-old chicks are key efficiency and profitability factors in poultry production [2]. Yet, the poultry industry heavily depends on imported inputs, including feed ingredients, supplements, day-old chicks, and vaccines. This reliance renders the industry vulnerable to inflation caused by foreign exchange fluctuations. Limited access to credit for agricultural production is another key challenge due to the perceived riskiness of agriculture in the country, deterring financial institutions from providing adequate support [13].

Within Ghana's agricultural policy landscape, poultry sector development may have been on the periphery. The poultry sector has historically not been considered a government priority, resulting in a lack of proactive policies specifically designed to support its growth [6]. Consequently, there is a dearth of proactive policies specifically aimed at supporting the growth of the poultry sector. Noting the significant difference that trade policies such as import bans and increased tariffs play in agricultural development, coupled with the underachievement of implemented policies in Ghana, Boimah et al. [2] called for a holistic assessment of policy interventions targeted at restricting imports to incentivise local poultry production due to the complexity of the problem. Moreover, Boimah et al. [2] emphasised the need for a participatory approach that allows the different stakeholders in the poultry value chain to collectively resolve the problems facing the industry. However, rallying diverse stakeholders with conflicting objectives proves to be a significant hurdle. Each actor in the value chain operates within their own realm, making it challenging to engage them in a unified effort to address problems beyond their immediate purview. Consequently, interventions proposed for isolated problems frequently overlook their potential unintended consequences.

The interactions of systemic problems at different nodes of a value chain produce archetypes that can serve as a guide for the implementation of effective solutions to the problems [14,15]. Without a comprehensive understanding of the dynamics at play within the system, well-intended interventions risk exacerbating existing issues or creating new ones altogether. Therefore, adopting a participatory group model building approach, this paper aimed to examine the system archetypes emerging from the inherent industry-level and farm-level problems in Ghana's poultry sector.

This paper makes two key contributions. First, the assessment of systemic challenges of Ghana's poultry sector provides stakeholders with a comprehensive overview of how the impacts of single-objective interventions they adopt reverberate throughout the entire poultry industry, thus enabling a thorough review of strategies. This increased awareness enables the design of more robust strategies for addressing challenges in the poultry industry. Second, the findings demonstrate that collaborative problem-solving approaches, which prioritise the collective well-being of the entire poultry industry ecosystem, are indispensable for devising sustainable solutions to drive the growth of the poultry sector in Ghana.

## 2 Methodology

A qualitative system dynamics modelling approach is adopted via a group model building (GMB) process [16] to map out the causal linkages among different causes and consequences of systemic problems at both farm and poultry-industry levels. The complexity of decision-making processes in agricultural value chains necessitates the use of a technique that retains qualitative attributes of traditional value chain analyses [17]. The group model building process employed in this paper offers the full participation of stakeholders and promotes stakeholders’ ownership of the results obtained [18].

In system dynamics modelling, causal loop diagrams (CLDs) are simplified diagrammatic representations of the causal relationships among different interacting variables that shape the system structure influencing the system behaviour [19]. CLDs are useful for capturing complexity and analysing policy interventions [20]. The building block of the CLD is the relationship between two variables, which can be characterised as either positive or negative. A positive causal relationship, denoted by a positive polarity, occurs when an increase in one variable leads to an increase in another variable, while a negative causal relationship (denoted by negative polarity) indicates an inverse relationship between the variables. The interaction of various positive and negative causal relationships within a unidirectional closed circuit gives rise to a feedback loop, which can be either reinforcing or balancing.

### 2.1 Data

Officers from the Ministry of Food and Agriculture and the Ministry of Rural Development in Ghana were consulted to identify and recruit workshop participants. Emphasis was on poultry industry actors and others with knowledge and experience in various aspects of the sector, and who were willing and available to engage in the group model building process. A reference stakeholder group was constituted that reasonably represents the perspectives and interests of the commercial poultry sector in Ghana. Members of this group were purposively selected (using a snowballing method) to cover areas including the production of layer and broiler chickens, private veterinary service and other input provision, livestock/veterinary extension services, and academia.

**Fig. 1:**
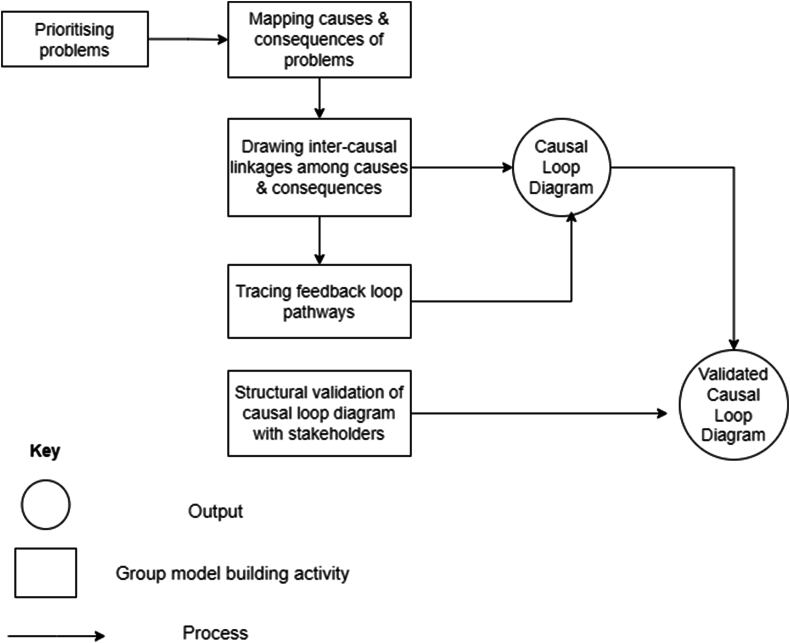
The group model building process.

Fifteen individuals comprising a diverse stakeholder group representing different segments of Ghana's commercial chicken value chain participated in the group model building process. The stakeholders included five people recruited as poultry producers, two input suppliers, three day-old chick agents, two product (egg) aggregators, and three officials from the Ministry of Food and Agriculture's (MOFA) Animal Production and Veterinary Services departments. However, some stakeholders played more than one role (e.g., producer and aggregator, producer and input supplier). Participants in the GMB process included representatives from national, regional, and theme-specific (e.g., women-focused) poultry associations. In addition to the GMB participants, three extension officers, from regional Livestock Departments, were involved as resource persons. These resource persons were observers who assisted with capturing data during the GMB sessions, and with providing context from regions not adequately represented in the proceedings. The GMB processes, implemented in a three-day workshop, are illustrated in Fig. 1.

**Fig. 2:**
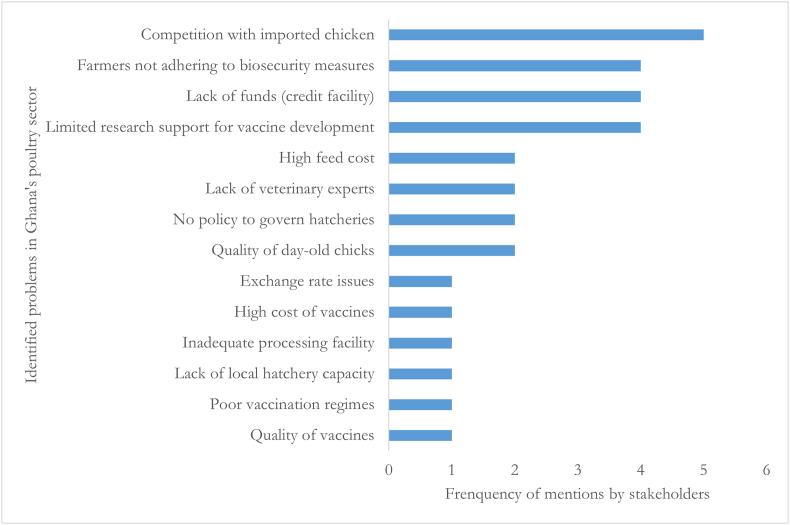
The prioritised systemic problems.

The first step of the GMB process involved identification and prioritisation of key problems in the poultry industry. Stakeholders brainstormed to generate a collective list of 14 problems following which they individually and anonymously selected their four topmost relevant problems, as shown in Fig. 2. Through a facilitated group discussion, the stakeholders identified and mapped the causes and consequences of each prioritised problem using CLDs.

The CLDs for the top four problems prioritised by all participants were mapped out in four separate groups that is one group per problem, led by the research team members including a lead facilitator, modeller, note takers, and a process coach [21]. The CLDs for the problems that emerged as the four most frequently prioritised by participants were mapped out in four separate groups, that is one group per problem. The mapping activity was led by the research team members that included a lead facilitator, a modeller, a process coach, and four note takers (one for each group). Four prioritised problems emerged from the prioritisation exercise with stakeholders. These were the following: (*i*) inadequate funding for vaccine research and development; (*ii)* lack of access to finance; *(iii*) the impact of input and output imports; and (*iv*) low adherence of biosecurity measures. The mapped CLDs were then consolidated by the modellers in the research team and visuals of the emergent CLDs presented to the stakeholders using the Stella Architect software. Subsequently, stakeholders validated the consolidated CLDs as an accurate representation of their conceptualisation of the identified systemic problems. The emerging system archetypes were examined based on the validated CLDs.

## 3 Results

The causal loop diagrams (CLDs) for each prioritised problem are presented and discussed in this section while highlighting the key feedback loops in the CLDs. Also, the key emerging system archetypes are explored by consolidating all CLDs for the prioritised problems. By delineating the focal implementers of interventions as the system boundaries, this paper discusses the intended and unintended consequences of stakeholders’ actions that give rise to the system archetypes.

### 3.1 Inadequate funding for vaccine-related research

**Fig. 3:**
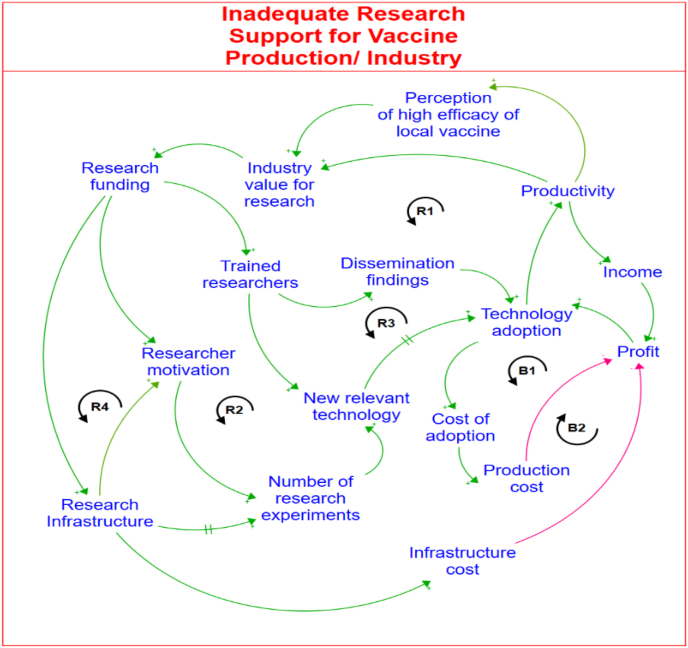
The causal loop diagram for limited research funding problem.

The results of the mapped out CLD on inadequate funding for vaccine-related research are shown in Fig. 3. The CLD provides insights into the pivotal role of research in fostering growth within the poultry industry, particularly emphasising the need for adequate funding to drive indigenous research innovations in vaccine production to combat poultry disease. The results indicate that increased funding for vaccine-related research will catalyse the training of more researchers, thereby boosting human capacity development in the relevant local research institutions. This, in turn, stimulates an increase in novel vaccine technological advancements aimed at enhancing on-farm productivity. As farmers embrace and implement these innovative technologies, their productivity increases, creating an increased value and demand for further research endeavours. Consequently, a reinforcing feedback loop (*R1*) perpetuates a virtuous cycle wherein increased industry investment in research is stimulated by the growing recognition of its value in driving the growth of the poultry industry.

Moreover, the results of the mapped out CLD show another reinforcing feedback loop pathway (*R2*) that links increasing research funding with increased researcher motivation to promote an increase in the number of research experiments conducted and the development of innovative technologies. The results from the CLD indicate that as these innovations are introduced and adopted by farmers, their productivity levels will increase to further strengthen the necessity for sustained investment in research. The reinforcing feedback loop (*R2*) highlights the relationship between research funding, technological innovation, and enhanced agricultural productivity within the poultry industry.

Also, the results of the CLD show that *ceteris paribus*, the increased availability of research funding will encourage a surge in the training of new researchers and facilitate widespread dissemination of research findings, which can lead to increased technology adoption among farmers to boost productivity levels. This chain of impacts creates a reinforcing feedback loop (*R3*). As productivity rises, the perceived value of research within the industry increases, and this will prompt further investments in research endeavours, as depicted by the reinforcing feedback loop (*R4*). This reinforcing feedback loop also represents a virtuous cycle that reinforces a mutually beneficial relationship between research funding and industry support, driving ongoing advancements and innovation within the sector.

However, the results indicate that within the CLD lies two balancing feedback loops that emphasise the importance of evaluating the cost-effectiveness of research innovations both at the industry and farm levels. The mapped out balancing feedback loop (*B1)* indicates that costly research innovations will reduce the profit of the research firm. Similarly, an increased cost of adopting innovative technologies increases production costs for farmers, thereby constraining attainable profits, as illustrated by the balancing feedback loop (*B2*). Consequently, research innovations that are not competitively priced may face reluctance from farmers to adopt them.

### 3.2 Farmers’ lack of access to credit

**Fig. 4:**
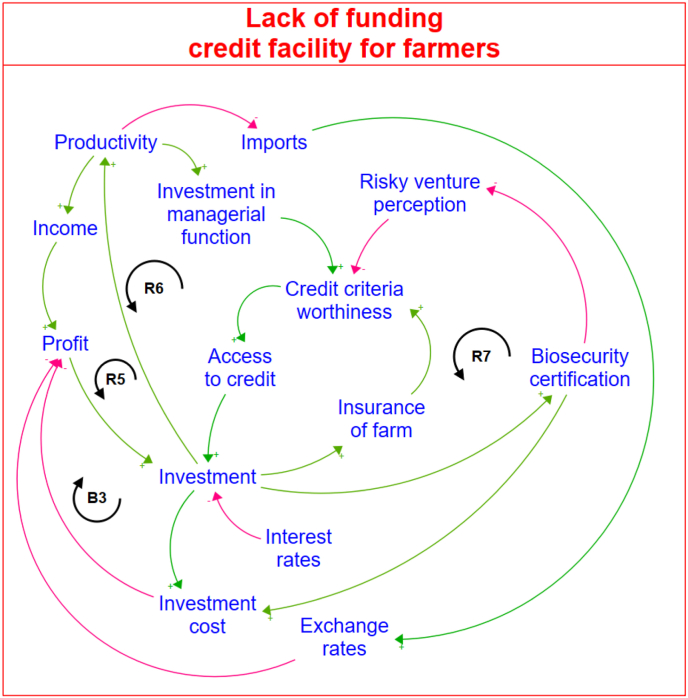
The causal loop diagram for farmers' lack of access to credit problem.

Results of the mapped out CLD in Fig. 4 illustrate the impact of poultry farmers' access to credit, particularly by highlighting the reinforcing feedback loop (*R5*), which shows how enhanced managerial capabilities among farmers contribute to increased creditworthiness, facilitating improved access to credit facilities. The reinforcing feedback loop (*R6*) shows how access to credit increases on-farm investments, improves farm productivity, and subsequent reinvestment in enhancing managerial functions. While access to credit is linked to higher on-farm profits, the balancing feedback loop (*B3*) indicates that the high investment cost of managerial functions can constrain other farm investments. Therefore, there is a need to seek cost-effective solutions to enhance a poultry farm's creditworthiness.

In the CLD, insuring poultry farms is proposed as a strategy to enhance the farm's creditworthiness. However, there is a feedback loop between access to credit and farmers' willingness to insure their farms. The reinforcing feedback loop (*R7*) in the CLD indicates that farm creditworthiness will be enhanced through the adoption of robust biosecurity measures to mitigate perceived agricultural risks.

### 3.3 Low adherence of biosecurity measures at the farm level

**Fig. 5:**
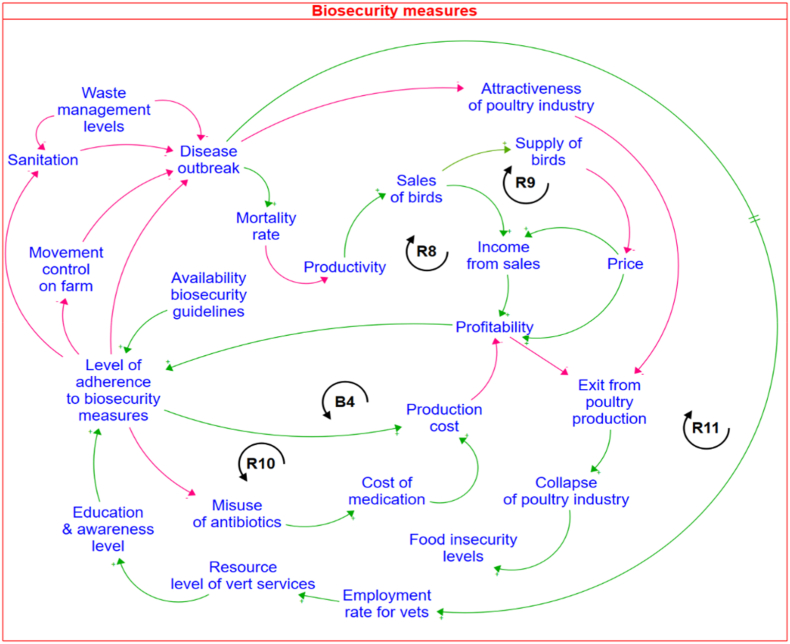
The causal loop diagram for the low adherence of biosecurity measures problem.

Results of the mapped out CLD in Fig. 5 highlight the benefit of adopting biosecurity measures. Both farm-level and industry-level factors that either constrain or enable the adoption of biosecurity measures are also detailed in the CLD. At the farm level, the reinforcing feedback loop (*R8*) describes how the benefit of adhering to biosecurity measures, traced in *R7,* sustains the reinvestment in biosecurity measures. Additionally, the reinforcing feedback loop (*R9*) highlights the potential market opportunities that arise when biosecurity measures are implemented during disease outbreaks. Such outbreaks typically result in increased on-farm mortality rates, leading to decreased productivity at farm level.

At an aggregate level, reduced productivity is linked to a decrease in market supply, driving an increment in the price. Farmers who adopt biosecurity measures are protected from disease outbreaks, preventing declines in productivity. Consequently, these farmers can seize the resulting price increases to enhance their profits. However, the balancing feedback loop (*B4*) highlights the cost implication for adhering to biosecurity measures. When farmers fail to generate sufficient profit from these measures, they may find themselves trapped in a vicious cycle, as depicted by the reinforcing feedback loop (*R10*), resorting to the misuse of antibiotics as a cheaper alternative.

An unfortunate situation illustrated in the CLD pertains to the reactive nature of the government's policies. While educating and raising awareness of chicken producers about the benefits of implementing biosecurity measures is recognised as an effective approach to improve adherence at the farm level, there remains a critical need to strengthen the veterinary workforce. However, the likelihood for the government to employ more veterinary officers only increases when there is a disease outbreak. The government's reactionary stance creates a vicious cycle depicted by the reinforcing feedback loop (*R11*). At the industry level, disease outbreaks diminish the attractiveness of poultry production businesses, prompting producers to exit the industry, and subsequently leading to its collapse of the industry, which in turn exacerbates food insecurity. In response, importing poultry products becomes a shortcut solution to address the systemic policy failure. However, this approach leads to unintended consequences, which will be discussed in detail in the next section.

### 3.4 The impact of inputs and meat chicken imports

**Fig. 6:**
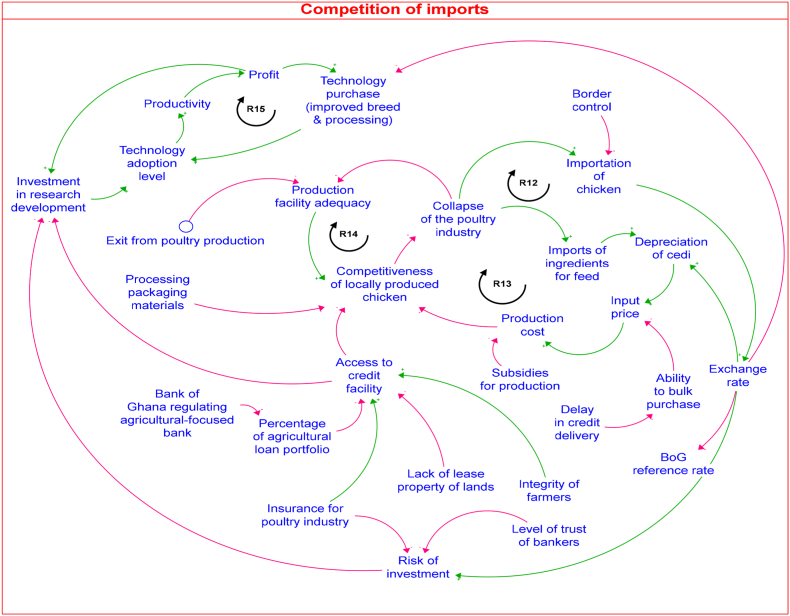
The causal loop diagram for the competition of imports problem.

Results of the mapped out CLD in Fig. 6 show how the heavy dependence on imports poses significant food and nutrition security risks for Ghana, highlighting the urgent need for strategies to enhance domestic production capacity and reduce reliance on foreign sources. Particularly, two reinforcing feedback loops (*R12 and R13*) highlight the vicious cycle of excessive import dependence. Importing inputs for domestic production escalates production costs and diminishes the competitiveness of locally produced chicken meat, precipitating the industry's collapse and necessitating increased imports to sustain demand. The influx of cheaper imports leads to a further decline in the competitiveness of the local production, as illustrated by the reinforcing feedback loop (*R14*).

In the same vein, the reinforcing feedback loop (*R13*) shows how the collapse of the local industry will lead to increased imports, which will affect the exchange rates leading to a depreciation of the local currency and subsequently an increase in production cost.

### 3.5 Emerging problem archetypes

The emerging archetypes within the mapped CLDs are discussed in this section, with the implementing actor serving as an indication for the system boundary. System archetypes serve as generic structures that offer insights into the conceptualisation of complex problems [14]. Three problem archetypes emerge from the interacting CLDs.

First, an underachievement archetype, specifically the limit to growth emerges when reinforcing feedback loops *(R1, R2, R3*, and *R4)* related to technology adoption due to increased research funding interact with the balancing feedback loop (*B4*) concerning the non-adherence to biosecurity measures as a cost-cutting strategy at the farm level. The underachievement archetype is so called because it depicts a situation in which reinforcing events ensure that the industry consistently fails to reach expected potential. Thus, the expected growth benefits from the technology adoption are not realised due to the growth limitations caused by non-adherence to biosecurity measures. In the CLD, the balancing feedback loop predominates when there is limited education and sensitisation to raise awareness, attributed to the government's reactionary policies regarding the recruitment of veterinarians. This archetype draws attention to the need to consider the cost-effectiveness of the adoption of new technological innovations from the purview of the farmer. The funding of research innovations that ends up being costly on the market would be detrimental in the long run.

Second, the out-of-control system archetype emerges when the balancing feedback loop (*B4*) revolving around the misuse of antibiotics as a cost-cutting strategy due to non-adherence to biosecurity measures interacts with the vicious reinforcing feedback loop (*R10*), which revolves around the benefits of reduced production cost for not adhering to biosecurity measures when there is no disease outbreak. The out-of-control system archetype describes a situation in which an intervention or action intended to address a problem fails, ultimately leading to the emergence of a new problem. This interaction exacerbates into a delayed reinforcing feedback loop (*R13*), originating from the industry's collapse and the consequential surge in chicken imports into the country. The misuse of antibiotics represents a “*fix that fails*” solution to the systemic problem of non-adherence to biosecurity measures. The out-of-control archetype arises because the intended control to reduce production costs through non-adherence to biosecurity measures fails, resulting in reduced productivity and the eventual collapse of the industry.

Third, the relative achievement archetype emerges from the reinforcing feedback loop (*R13*), which centres around higher imports of chicken to fill gaps in domestic supply *(R14*). The relative achievement archetype occurs when the resolution of food insecurity through cheap imports ultimately causes the collapse of the broiler industry. While importation initially provides increased access of consumers to affordable animal protein in the short term, the unintended consequence of cheaper imports is that the industry is significantly deteriorated over time, adversely affecting the entire poultry industry.

A specific manifestation of the relative achievement archetype is the “*success to the successful*” scenario, wherein midstream actors in the poultry value chain engaged in the importation of chicken become more prosperous at the expense of the entire industry. To address this archetype, regulations aimed at controlling the influx of cheaper imports are imperative. Therefore, the stakeholders propose the implementation of border controls to curb the influx of cheaper imports, along with an increase in import tariffs, as effective solutions. These measures are crucial to safeguarding the stability and sustainability of the domestic poultry industry while ensuring a level playing field for all stakeholders involved.

### 3.6 Discussion

#### 3.6.1 Research funding and access to credit

The findings from the group model building process highlight government funding as a major source of research funds in Ghana. Kusi et al. [22] emphasised the importance of government funding for research in revitalising the local poultry sector as a financially sustainable alternative to poultry imports. The findings of the mapped out CLD show that the sustainability potential of research funding is highlighted within the reinforcing feedback loop (*R3*). However, without strategic allocation and effective management, research funding may not lead to the training of new researchers. Consequently, there will be no dissemination of research findings (which require clear strategies, actions, and dedicated budgeting), leading to low research technology adoption rates, and ultimately reduced productivity. Therefore, the involvement of research managers in decision-making processes concerning research funding is crucial.

Furthermore, the findings identified access to credit and the timely provision thereof as crucial factors influencing technology adoption and the technical efficiency of poultry farmers. Factors that influence farmers' access to credit tend not to favour smallholders and neophytes. Experienced farmers and farmers with large farm sizes are more likely to access credit [8]. Also, disparities in credit accessibility particularly concerning gender have been reported; with male producers being more likely to access credit compared to their female counterparts [23]. Despite these disparities, the reinforcing feedback loop (*R6)* in the CLD suggests that establishing managerial functions provides an objective mechanism to determine a farm's creditworthiness; thus, granting equal access to finance regardless of the farmer's gender and operation size.

The inference drawn from the CLD suggests that farmers should prioritise establishing effective managerial systems before pursuing credit. A potential strategy to enhance poultry production performance, as proposed by Dziwornu & Assefuah [24] and Quaicoo & Bannor [25], involves separating ownership from farm management. Additionally, collaborating with fellow producers and financial institutions through savings initiatives can increase the likelihood of obtaining credit. Acheampong [26] underscores the increased likelihood of commercial poultry farms surviving when there are established ties to financial institutions. Moreover, contract farming been also has proposed as a business model to enhance producers’ access to services that may otherwise be inaccessible due to credit constraints [27].

The findings of this paper illustrate how high interest rates can restrict access to credit, highlighting the need for policy interventions to regulate and facilitate credit accessibility. Interestingly, an unintended consequence of facilitating access to credit, beneficial to the entire poultry industry, is noted by Apike et al. [28], who observed an increased likelihood of imported chicken distributors to embrace poultry products produced and processed domestically. Thus, financial institutions facilitating credit access have a role to play in stimulating interest in investment by midstream poultry value chain actors to revamp the local poultry sector.

#### 3.6.2 Promoting adherence of biosecurity measures

Diseases pose a significant risk to poultry producers' productivity [13,29]. As noted by Okata et al. [30], farmers’ income has a feedback effect on adherence to vaccination schedules. The findings of this paper show that farmers will opt for cheaper ways to curtail the impact of disease outbreaks. Hence, the regulation of antibiotic usage is needed due to its impact on poultry meat quality [31]. Effective policies aimed at ensuring the judicious use of antibiotics are essential to safeguard both public (human and animal) health and poultry industry sustainability.

Policy initiatives promoting the implementation of biosecurity certification for farms have been recommended as a mechanism to facilitate access to credit. However, this paper's findings indicate that the existence of the balancing feedback loop (*B3*) implies that the cost associated with implementing biosecurity measures at the farm level may offset the benefits derived from the reinforcing feedback loops associated with access to credit. Consequently, the effectiveness of the intended policy on biosecurity certification to secure credit is limited when it fails to translate into increased revenues from farmers’ downstream activities.

#### 3.6.3 Addressing the impact of imported poultry inputs and products

Ghana's poultry industry heavily relies on imported inputs such as day-old chicks, vaccines, and poultry feed to sustain domestic production, while also depending on imported chicken to meet the increasing consumer demand for chicken meat [32]. Despite the potential for large-scale production to improve the industry's productivity, small-scale producers dominate Ghana's poultry sector, contributing to stagnation in its growth [12]. There are local hatcheries in the country that produce day-old chicks at a lower cost compared to imported ones [33]. While some farmers prefer imported chicks due to perceived higher quality, the widespread availability of these imports ultimately leads to the collapse of local hatcheries, rather than encouraging improvements in the standards and quality of locally hatched chicks. Additionally, due to the high production cost and the stiff competition faced by broiler producers in Ghana, there has been a shift from broiler production to layer production [23], creating a significant deficit in the local supply of chicken meat.

Moreover, feed cost and the cost of day-old chicks constitute the majority proportion (∼70 %) of total production cost [34]. Consequently, the findings of this paper show that producers actively seek strategies to mitigate production costs. One such strategy is the more frequent use of imported day-old chicks in domestic poultry production. Chicks from this source are, perceived to have superior performance than locally hatched day-old chicks with at least some of the perception stemming from expectations around the management of incubation facilities [33]. However, this paper's findings show that the preference exacerbates the reliance on importing day-old chicks, further perpetuating the vicious cycle. As highlighted by Baagyere et al. [35], inadequate hatcheries within the country remain a significant bottleneck in the poultry industry's development, resulting in approximately 80 % of the total demand for poultry products (equivalent to 400,000 tonnes) being met through imports.

Extant studies have proposed market-oriented solutions to enhance domestic poultry production in response to the rising concerns over imports, with production-oriented strategies receiving comparatively less attention. According to Asante-Addo and Weible [36,37], there is a general stated preference for locally-produced chicken in Ghana. However, the revealed preference showed that factors like price, packaging convenience, and availability influence the stated preference. Hence, most consumers in Ghana patronise imported chicken meat. Although there is a strong stated preference for locally-produced chicken, there is a higher production cost and a consequential higher price for locally-produced chicken [38]. Thus, Apike et al. [28] contend that employing marketing channels and strategies such as packaging poultry meat in smaller portions, akin to those used for imported chicken, could stimulate interest in locally produced poultry meat. Furthermore, it has been observed that aligning the price of locally-produced poultry meat with that of imported chicken significantly impacts the willingness of midstream actors to distribute locally produced chicken in the value chain [28].

In their assessment of the impact of importation, Dieye et al. [39] reported that 25 % of hatcheries are underutilised in Ghana. However, proponents of increased imports have noted the roles of imports in helping sustain the food and nutrition security needs of poor households in the country. Knößlsdorfer and Qaim [40] argued that without cheap imports of chicken meat, poor households would not be able to afford chicken meat. However, this paper's findings suggest that the import proponents take a narrow view of the impact without considering the cascading impacts of growing domestic production. The potential impact on the input sector needs to be accounted for to make a holistic appraisal of the impact of imports as shown in the findings of this paper.

The imposition of import tariffs is one of the approaches that has been used to reduce importation. However, it has the potential of leading to an increase in domestic prices [12]. A complete ban on chicken meat imports to Ghana may be unrealistic in the short term due to likely impacts on food security, as demand will go largely unmet. However, a strategic increase in the current 35 % import tariff could reduce the market share of imported chicken and help boost the market share of domestically produced chicken. Senegal is a prime example that Ghana can learn from, where an effective ban on imports resulted in the growth of the domestic poultry industry [12]. However, the prominence of large-scale producers is a significant factor contributing to the effective implementation of the ban.

Other strategies espoused are demand-focused and pertain to using advertisement of the freshness of domestic chicken as a credence attribute and the smaller packaging of domestic chicken meat to increase demand [36,37]. As household income increases, the probability of consuming domestic chicken instead of imported chicken increases, highlighting the market potential of locally-produced chicken meat.

## 4 Conclusion

In this paper, systemic problems affecting Ghana's poultry industry were examined using a participatory group model building process. Based on the mapped out causal loop diagrams, three problem archetypes emerged, including the underachievement, out-of-control (specifically, fix that fails), and relative achievement (specifically success to the successful) system archetypes.

Four solutions are proposed to address the emerging problem archetypes. First, for the underachievement archetype relating to technology adoption, the solution archetype lies in ensuring that new technological innovations are affordable and cost-effective to incentivise farmers to increase adoption. Second, for the out-of-control (fix that fails) archetype related to the misuse of antibiotics, the implementation of biosecurity certification to enhance the creditworthiness of poultry farms that ensures market-oriented benefits for farmers is recommended as a solution archetype. Complementarily, the establishment of effective managerial systems at the farm level via the separation of farm ownership from farm management can be prioritised before pursuing credit acquisition. Finally, the relative achievement archetype can be counteracted by strategically imposing tariffs within value chain segments, particularly upstream, to safeguard domestic production industries.

This paper's findings show that the government's reactionary policy to strengthening the workforce of the veterinary department is detrimental to the industry, creating a vicious cycle that perpetuates the low adherence of farms to biosecurity measures. Moreover, stakeholders show conflicting views on the biosecurity certification scheme's potential to simultaneously reduce antimicrobial misuse in the poultry industry and improve farm creditworthiness. Consequently, government officials should reassess the scheme's scope and potential advantages.

This paper provides insights into the systemic challenges facing Ghana's poultry industry. The conclusions drawn are qualitative and lack precise quantification of the impacts of proposed solutions on systemic problems. Hence, future studies would focus on assessing the effectiveness of these solutions to validate their ability to achieve the intended outcomes.

## CRediT authorship contribution statement

**Joshua Aboah:** Writing – review & editing, Writing – original draft, Visualization, Validation, Software, Methodology, Investigation, Formal analysis, Data curation, Conceptualization. **Dolapo Enahoro:** Writing – review & editing, Writing – original draft, Supervision, Resources, Project administration, Investigation, Funding acquisition, Conceptualization. **Charles Mensah:** Writing – original draft, Resources, Methodology, Investigation, Conceptualization. **Nana Adwoa Agyemang:** Writing – original draft, Resources, Methodology. **Ebenezer Kondo:** Writing – original draft, Methodology, Conceptualization. **Desmond Ayertey:** Writing – original draft, Resources, Methodology.

## Declaration of competing interest

The authors declare no conflict of interest that may be affected by the research reported in the enclosed paper.

## Data Availability

Data are qualitatively mapped out in the causal loop diagrams
